# HCSD: the human cancer secretome database

**DOI:** 10.1093/database/bav051

**Published:** 2015-06-13

**Authors:** Amir Feizi, Amir Banaei-Esfahani, Jens Nielsen

**Affiliations:** ^1^Novo Nordisk Foundation Center for Biosustainability, Department of Biology and Biological Engineering, Chalmers University of Technology, SE-41296 Göteborg, Sweden, ^2^Novo Nordisk Foundation Center for Biosustainability, Technical University of Denmark, Fremtidsvej 3, DK-2970 Hørsholm, Denmark and ^3^Novozymes A/S, Krogshoejvej 36, 2880 Bagsvaerd, Denmark

## Abstract

The human cancer secretome database (HCSD) is a comprehensive database for human cancer secretome data. The cancer secretome describes proteins secreted by cancer cells and structuring information about the cancer secretome will enable further analysis of how this is related with tumor biology. The secreted proteins from cancer cells are believed to play a deterministic role in cancer progression and therefore may be the key to find novel therapeutic targets and biomarkers for many cancers. Consequently, huge data on cancer secretome have been generated in recent years and the lack of a coherent database is limiting the ability to query the increasing community knowledge. We therefore developed the Human Cancer Secretome Database (HCSD) to fulfil this gap. HCSD contains >80 000 measurements for about 7000 nonredundant human proteins collected from up to 35 high-throughput studies on 17 cancer types. It has a simple and user friendly query system for basic and advanced search based on *gene name*, *cancer type* and *data type* as the three main query options. The results are visualized in an explicit and interactive manner. An example of a result page includes annotations, cross references, cancer secretome data and secretory features for each identified protein.

**Database URL:** www.cancersecretome.org.

## Introduction

Cancer is currently seen as a cluster of complicated diseases with increasing prevalence globally (1). Understanding and curing cancer have entered a new phase with the advent of next generation sequencing and advanced proteomics (2). In particular, recent advances in both accuracy and scale of the measurements in proteomics using label-based methods (such as SILAC and iTRAQ) have revolutionized oncoproteomics (3). The cancer secretome, as a newly established subdiscipline of oncoproteomics, involves the detection, quantification and characterization of the secreted proteins (such as cytokines, growth factors etc.), shedome (shed receptors and proteases) and extracellular matrix components of a given type of cancer cell at a specific time point (4, 5). Many secreted proteins are linked to the hallmarks of cancer which are reliant on cell–cell adhesion and signaling (6, 7). Much analysis supports how these proteins in the tumor microenvironment control and regulate the cancer cell invasion and metastasis (8–11). Along with this, soluble factors in cancer secretome are promising for novel biomarkers and therapeutic targets for different types of cancers (6, 12–16). Accordingly, there has been increasing number of studies to analyze the cancer secretome resulting in rapid growth in data generation. For example, Wu and coworkers identified candidate serological biomarkers for various cancer types based on secretome analysis of 23 cancer cell lines (17). From 4584 nonredundant proteins identified in these cancer cell lines, they suggested between 6 and 137 marker candidates selective for each tumor type and 94 potential pan-cancer markers (proteins secreted by most cancer cell lines) and they verified several of the identified protein biomarkers (17). There are many other examples of the same kind of studies that have provided large amounts of data to be publically available (18–20). However, the lack of a specific database for cancer secretome data challenges researchers in the field to query community knowledge in terms of the time and efficiency. Therefore, designing a systematic and organized database to manage large volumes of unstructured cancer secretome data is in demand. To fulfil this important gap, we designed the Human Cancer Secretome Database (HCSD), a dynamic database with interactive web interface that provides the researchers with the opportunity to explore their protein of interest against the publicly available data on the human cancer secretome. HCSD has a simple and user-friendly query system for basic and advanced searches based on *gene name*, *data type*, and *cancer type* as the three main query options. The result pages are explicit and intractable. An example result page includes annotations, cross references, cancer secretome data and secretory features for each protein. Developing HCSD is an important bioinformatics solution to boost research in cancer secretome and tumor microenvironment.

## Materials and methods

### Data collection and preprocessing

To collect all relevant data from high-quality publications, a comprehensive literature survey was done searching the *Scopus* and *PubMed* database starting with the general keyword ‘cancer secretome’. To avoid accumulation of the false identifications that is frequent in proteomics data, we applied stringent selection criteria to filter out publications including: (i) to have standard workflow of one of the shotgun proteomics techniques (with biological and/or technical replications). (ii) Detailed description for each steps of the experimental design. (iii) Providing of all the parameters used in database searching and corresponding bioinformatics analysis. (iv) Having error estimation strategy (such as FDR). (v) Performing molecular/clinical validation experiment for the identified biomarkers. (vi) Providing supplementary detail information tables for identified portions in peptide and protein level. Applying these criteria total 35 high-throughput publications were selected as data source to collect the relevant data (see DATA SET menu in the web page). 

A major concern of any proteomic study is the FDR (Flase discovery rate) control to prevent from inflation of false identifications. To obtain reliable results, 1% FDR should be applied on peptide and protein levels. When merging distinct datasets, which were analyzed separately, one has to take special care to avoid inflation of the FDR. This has been previously done by Schaab *et al.* (21). However, to apply such techniques in merging proteomics datasets, corresponding *P* *values* for the reported fold changes are necessary. Unfortunately, most of the available data sets have not included the raw data or the *P* values in their released data sets. This was a big challenge in designing HCSD, and to overcome to that, we therefore carefully collected the data based on the cut-off FDR reported in each paper and we did exclude all the proteins above used the cut-off. Also, the query results designed to be based on each study so the user can compare the results from different studies on a particular protein of interest and decide based on major votes. In line with this, we also provided more technical information on identification such as PSMs (peptide-spectrum matches) and the number of the unique peptides match in the results pages for each query. Doing this while it is not yet possible to fully resolve the inflation of false identification resulting from combining independent studies, the results will be reported study wise so the user can assess the reliability of the results by checking other supportive information from each study.

**Figure 1.**
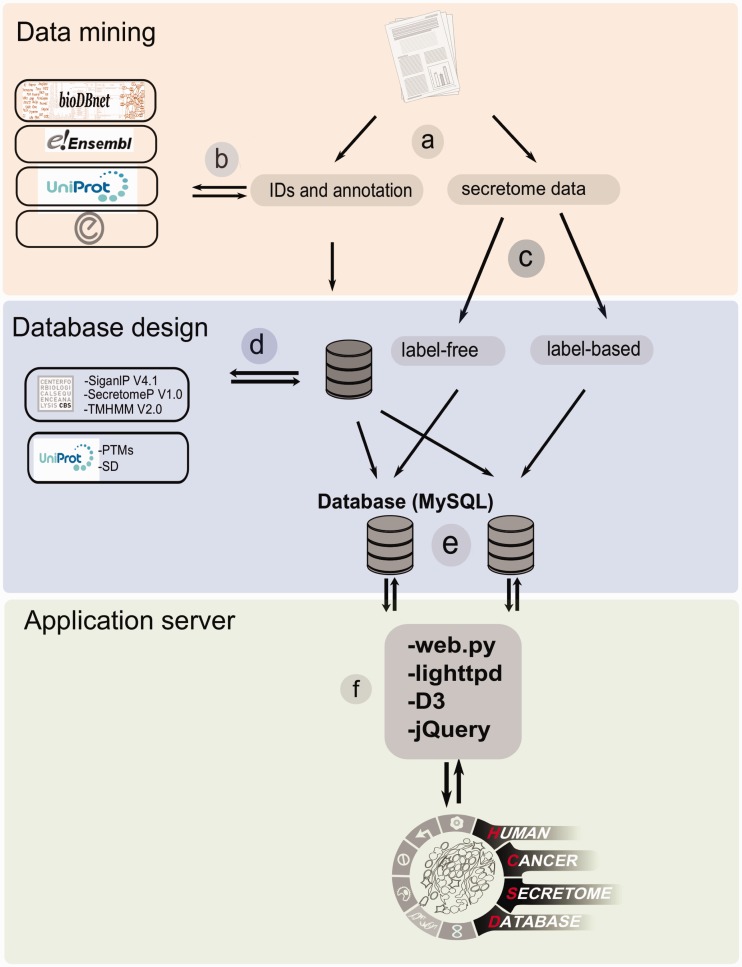
The workflow of HCSD design. (**a**) Appling the selection criteria, first all the cancer secretome data were collected and processed from literatures. (**b**) Then, I all the complementary annotation and cross references were obtainedfrom UniProt, Ensembl, bioDBnet and Entrez using thethe reported protein or gene IDs in the data tables. (**c**) Next, the secretory pathway features including signal peptide, transmembrane domains and nonclassic secretory proteins were predicted using CBS prediction servers (32–34). The secondary structures and PTMs information were retrieved from UniProt (35). (**d**) Based on the proteomics strategy used, the secretome data were divided into label-free and label-based studies. (**e**) The structured data tables used as input to MySQL to generate searchable data tables by end user (http://www.mysql.com/). (**f**) For the web server lighttpd is used to query the database (http://www.lighttpd.net/) and the web application and the interface were implemented using web.py (www.webpy.org), Javascript (http://en.wikipedia.org/wiki/JavaScript), jQuery (http://jquery.com/) and D3 (http://d3js.org/).

Next, the publications were categorized as *label-free* and *label-based* studies based on the proteomics techniques have been used to quantify the proteome. In *label-free* proteomics, the secretome of a specific cancer type is quantified without using a stable isotope containing compound and the peptide abundance is quantified by spectral counting. On the other hand, in *label-based* proteomics, stable isotope(s) is used for labeling and quantification of the peptides in the comparable samples. The *label-based* methods are less sensitive to the experimental biased than *label-free* methods (22, 23). Therefore, this categorization helps user to compare the results from two type of technology. Because of the difference in publishing the data from one paper to another, retrieving and processing data tables from various studies and merging them into a single database structure was time consuming. Missing information and the format of released data (PDF format) were also problematic in data collecting step. As most of the studies only report their data based on gene symbol or protein ID, for ID mapping, we used bioDBnet (24) to make data be searchable using different IDs in gene and protein levels. For each protein in the database the annotation data extracted from UniProt (25), ensemble (26) and Entrez (27) (Figure 1). An exclusive link for each record is provided to direct the user to its HPA cancer atlas (28) page. The HPA page provides the user with antibody-based protein profiling information for the protein of interest in 20 most common cancers. This allows user to compare the expression status of the collected proteins in HCSD based on quantitative methods against antibody-based staining data in HPA.

### Databasing and interface design

We used a MYSQL relational database (version 5.5.8) to design and query HCSD database. The web interface implemented by *webpy* (www.webpy.org), a python based web framework. The *web.py* is in the public domain and it has been used by Google App Engine. To our knowledge; this is the first implementation of it in designing a bioinformatics database.It is as powerful as *Django* (https://www.djangoproject.com/) while it is much simpler to implement. The *lighttpd* (http://www.lighttpd.net/) were used as a fast and open source web server which security, speed, compliance and flexibility are all its characteristics comparing to other competitors. In the query page, two dynamic and searchable tables for *label-free* and *label-based* data were designed using DataTables plug-in of jQuary (https://www.datatables.net/). The result pages benefit from high quality visualization techniques to present the cancer secretome data and secretory features. For visualization, Javascripts and D3 (www.d3.org) were used upon *web.py*. HCSD is available at www.cancersecretome.org.

### Querying HCSD

**Figure 2.**
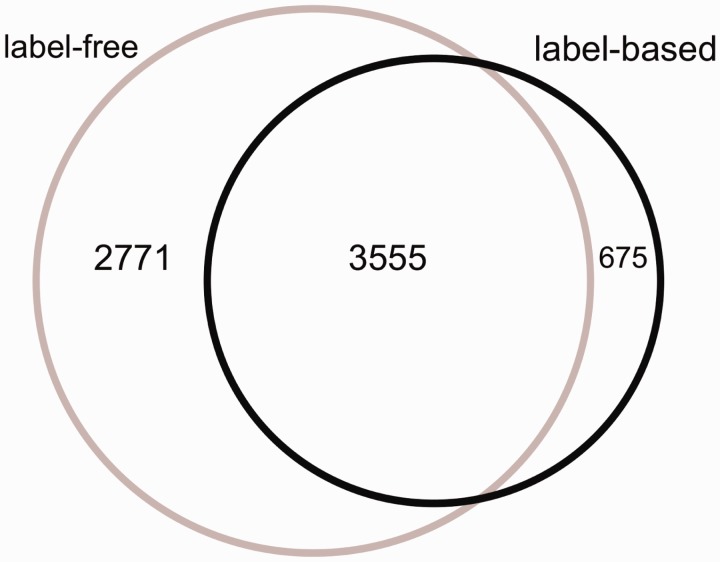
The Venn diagram of the proteins measured in label-free and label-based studies (35 publications).

**Figure 3.**
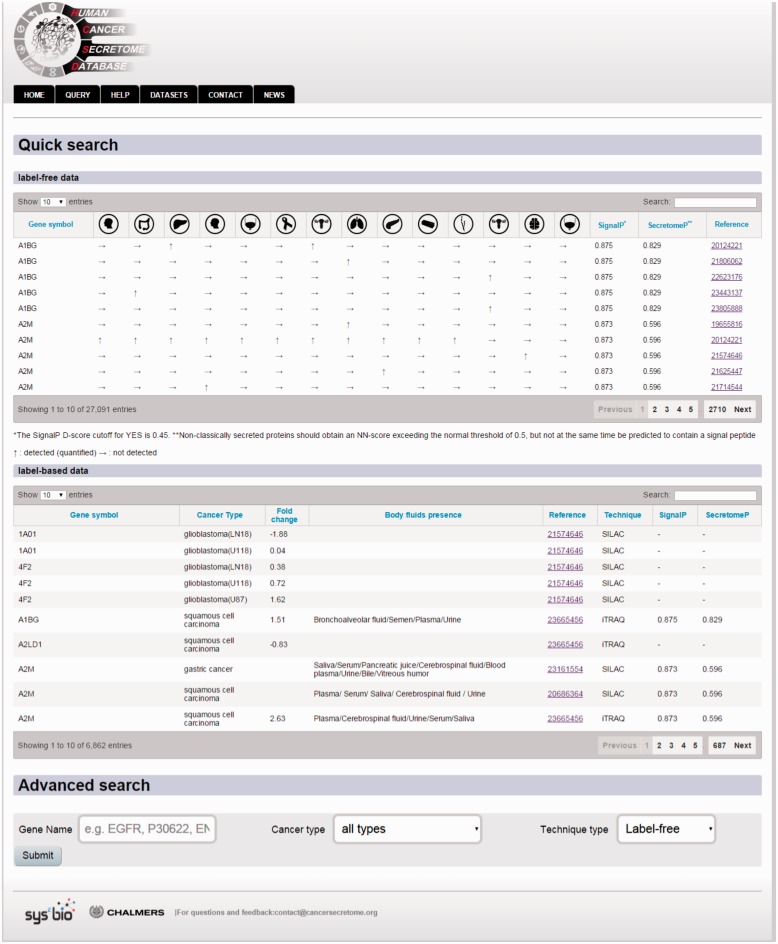
Example of the query page showing the search options.

**Figure 4.**
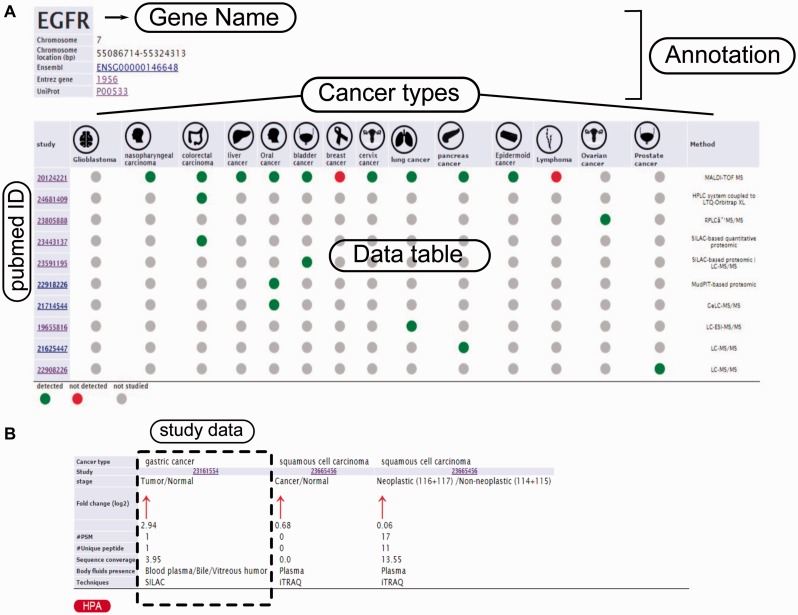
Example from the result pages for label-free and label-based studies. In the result page, the first section (**a**) provides the annotations such as gene name, description, chromosomal location and cross references ID to the Ensembl (http://www.ensembl.org/), Entrez (http://www.ncbi.nlm.nih.gov/), and UniProt (www.uniprot.org/). In case of label-free search, exploring all type of cancers will be visualized as a table with the cancer type icons in the header. The first column contains hyperlinked PubMed IDs. For each cancer type column, the protein of interest is detected (green spot), not detected (red spot) or not studied (grey spot). The last column specify the proteomics method used in the study. (**b**) In the case of label-based data, the result table header includes cancer types and the follow up information including the cancer stages, quantified fold change, number of the PSMs, number of the unique peptides, sequence coverage and body fluid presence will come as additional rows.

In order to query HCSD data, the user can start with quick search in the two interactive tables for the label-free and label-based data based on the gene of interest or information in other columns. Also, these tables are sortable for any columns of interest. We also designed an advanced query option for the user in order to query the protein/gene of interest to get more detail information. To do the advanced query, the user first needs to specify a gene symbol, UniProt or Ensemble gene ID in the query box. For example, if the target gene name is EGFR, the user can enter the EGFR in the query box (the first query field). The integrated autocomplete feature will let the user to choose the gene name or IDs in case of uncertainty. Next, the user has to select the cancer type of interest (or all the cancer types). The last option is to choose the data type which has three choices- the label-free, label-based and both options. Then, the user can submit the query to the server. The advanced query provides the user the possibility to combine various queries between the cancer types and quantification techniques. The result pages of label-*free* and *label-based* are similar in annotations and secretory features section (Figures 3 and 4), but they differ in secretome data results (Figure 2). For details explanation of the results pages see to the Figures 2–4.

**Figure 5.**
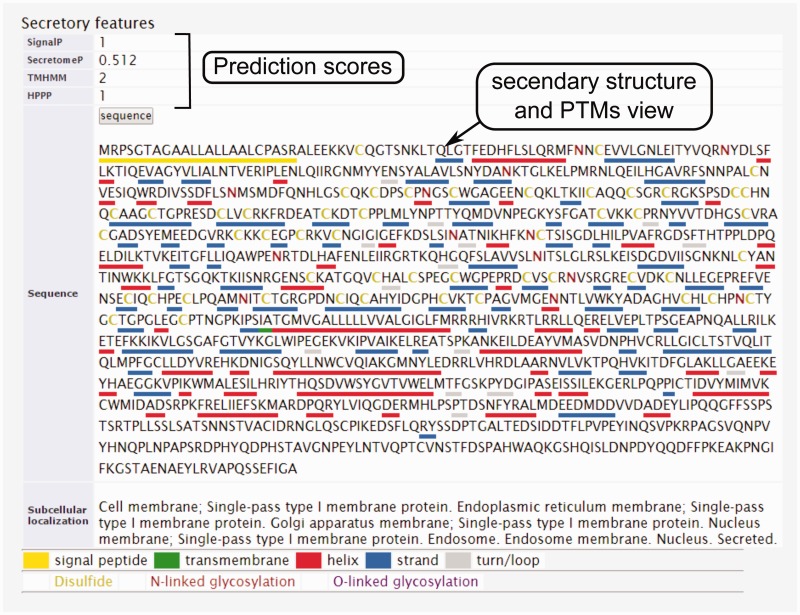
The secondary structure, secretory pathway features, subcellular localization and PTMs information. Querying both label-free and label-based studies, the second part of the result page is specified for the prediction scores of the secretory features and visualization of the PTMs and secondary structure information. The secretory features include scores of SingalP (33) (for signal peptide), TMHMM (for transmembrane domain) (32), SecretomeP (32, 34) (for nonclassical secretion), and HPPP (for human plasma membrane proteins) (36). The last row of the table shows the subcellular localization data. The PTMs are color coded. The color code legend for PTMs and secondary structure information will appear below the table.

## Results

### The structure of the HCSD

The HCSD structure was designed to fulfil four main goals (i) to provide a straightforward searchable depository for published data on different types of human cancer secretome, (ii) the ability to compare information across different secretome measurements (iii) to provide annotation; cross-references in both gene and protein level for each data points and (iv) prediction and visualization of the secretory features for each protein. Therefore, HCSD contains all the proteins (peptides) that are quantified so far to be (differentially) expressed in various cancer types secretome and at the same time provides annotation and predictions about their secretory type.

In eukaryotic cells, protein secretion is carried out either by the classic secretion pathway (having N-terminal signal peptide) or the non-classical pathway(s) (29, 30). It is valuable to know which processes the detected proteins potentially use for secretion in to the tumor microenvironment. Beside this, secretome analysis always is contaminated with proteins from cell debris or culture media that results in false identifications. To assist with these challenges, bioinformatics algorithms have been developed that can predict the secretory type of proteins from primary sequence based on signal peptide pattern, transmembrane domain or other motifs. These tools are extensively reviewed elsewhere (31). However, checking the reliability of the detection in secretome analysis is tightly depending on these tools, and therefore a secretory feature section is included in the results page for each protein query in order to give a summary of the predictions on signal peptide, transmembrane domain and nonclassical secretion signals using the most frequently used bioinformatics tools (Figure 1). Moreover, specific post-translational modifications (PTMs) are another characteristic of secretory proteins among which disulphide bonds and glycosylation sites (N-linked and O-linked) are the most specific. These information also can be visualized on protein sequence by the user in the result page (Figure 5).

An exclusive menu called ‘DATA SETS’ were designed which allows the user to get access and query the basic information about the publication used as data source. Each publication also has its own page which provides more details on the workflow and experimental design. The data set table provides hyperlinks to each publication PubMed page. The *study* column in the result page also directs the user to PubMed page of the corresponding publication.

### The statistics of the HCSD

From 87 496 total measurements stored in HCSD, ∼85% are derived from label-free on 14 cancer types. So far, the label-based cancer secretome analysis has been mainly performed on 5 cancer types (Supplementary Tables S1 and S2). The Lung cancer secretome is the most studied cancer and includes ∼11% of the total data (Supplementary Tables S1 and S2). From 7001 unique proteins in HCSD, 6326 are measured in label-free (with 1148 being transmembrane proteins) and 4230 being measured in label-based (with 534 transmembrane proteins).These two datasets share 3555 proteins (Figure 2). In general, most of the proteins detected in different cancer types secretome are secreted by nonclassical secretion pathways (Figure S1). In total, 1413 nonredundant proteins are detected to be secreted by classic secretion pathway in 14 cancer types from 21 label-free, while this number for nonclassical secreted proteins is 4,945. These numbers in label-based studies are 840 (classic) and 3409 (non-classic) proteins (Supplementary Tables S1 and S2). Most of the cancer secretome data was generated on cancer cell lines. In 35 publications used to design HCSD, 70 cancer cell lines were used to study the cancer secretome (Supplementary Table S3). In case that the authors did not include the cancer type of the cell lines they used, we included the corresponding cancer type.

## Discussion

How secreted proteins or peptides from cancer cells remodel the tumor microenvironment in favor of the metastasis is a pivotal research interest in the tumor biology. Cancer cell secretome profiling is a promising approach to find potential body fluid-accessible cancer biomarkers and therapeutic targets, however mining the increasing data from different labs is a big challenge which affects the efficiency of selecting useful candidates and results in the accumulation of redundant and false identified proteins. HCSD (www.cancersecretome.org) was developed as a database to store and query publically available human cancer secretome data to bypass these challenges. It provides the researchers to have access to all the high-throughput data from studies in this field together with the needed detail information in terms of the functional annotation and secretory type for each protein. It also allows exploring previously used workflows, cell lines, validated biomarkers and clinical surveys. HCSD can be used extensively by tumor biologist to find their target secreted factor in specific or various cancer types with all the annotations and sequence bioinformatics analysis of the primary sequence and secondary structure information of the target proteins. All this will facilitate the oncoproteomics studies in future.

## Supplementary Data

Supplementary data are available at *Database* Online.

Supplementary Data
