# Supplementary material for: HCSD: the human cancer secretome database
Source: Database (Oxford). 2015 Jun 13;2015:bav051. doi: 10.1093/database/bav051 (PMC4480035; doi:10.1093/database/bav051)
Supplement: Supplementary Data [file supp_2015_bav051_index.html]

HCSD: the human cancer secretome database — Supplementary Data 

# HCSD: the human cancer secretome database

## Supplementary Data

files

- Supplementary Data - zip file
