# Supplementary material for: HCSD: the human cancer secretome database
Source: Database (Oxford). 2015 Jun 13;2015:bav051. doi: 10.1093/database/bav051 (PMC4480035; doi:10.1093/database/bav051)
Supplement: Supplementary Data [file supp_bav051_suppl_data.zip › supplementary txt.doc]

**HCSD: the human cancer secretome database**

Amir Feizi^1, 2^, Amir Banaei-Esfahani^1^ and Jens Nielsen*^1, 2, 3^

^1^ Novo Nordisk Foundation Center for Biosustainability,Department of Chemical and Biological Engineering,Chalmers University of Technology, SE-41296 Göteborg, Sweden.

^2^ Novo Nordisk Foundation Center for Biosustainability,Technical University of Denmark, Fremtidsvej 3, DK-2970 Hørsholm

^3^ Novozymes A/S, Krogshoejvej 36, 2880 Bagsvaerd Denmark.

* To whom correspondence should be addressed. Tel: +46 31 772 3854; Fax: +46 31 772 3801; Email: nielsenj@chalmers.se

**Supplementary information**

**SUPPLEMENTARY TABLES**

**Table S1- The label-free statistics**

| **Cancer Type** | **Studies** | **Genes** | **Measurements** | **Secreted (classic)** | **Secreted Proteins (non-classic)** | **Transmembrane Proteins** |
| --- | --- | --- | --- | --- | --- | --- |
| Lung cancer | 6 | 4546 | 8719 | 998 | 3479 | 665 |
| Colorectal cancer | 5 | 5106 | 10482 | 1096 | 3978 | 812 |
| Glioblastoma | 3 | 1596 | 1871 | 338 | 1225 | 338 |
| head and neck squamous cell carcinoma | 4 | 4460 | 7632 | 882 | 3513 | 882 |
| Nasopharyngeal carcinoma | 1 | 4102 | 4489 | 842 | 3221 | 582 |
| Liver cancer | 1 | 4102 | 4489 | 842 | 3221 | 582 |
| Bladder cancer | 2 | 4139 | 4958 | 837 | 3230 | 837 |
| Breast cancer | 1 | 4102 | 4489 | 831 | 3199 | 831 |
| Cervix cancer | 1 | 4102 | 4489 | 831 | 3199 | 831 |
| Pancreatic cancer | 2 | 4175 | 5872 | 856 | 3249 | 856 |
| Epdermoid carcinoma | 1 | 4102 | 4489 | 831 | 3199 | 831 |
| Lymphma | 1 | 4102 | 4489 | 831 | 3199 | 831 |
| Ovarian Cancer | 2 | 2425 | 3076 | 630 | 1760 | 620 |
| Prostate Cancer | 1 | 4175 | 5872 | 856 | 3249 | 856 |
|  |  |  |  |  |  |  |
| Total | 21 |  | 75416 | 1413 | 4945 | 1148 |
|  |  |  |  |  |  |  |

**Table S2- label-based statistics**

| **Cancer Type** | **Studies** | **Genes** | **Measurements** | **Secreted (classic)** | **Secreted Proteins (non-classic)** | **Transmembrane Proteins** |
| --- | --- | --- | --- | --- | --- | --- |
| Lung cancer | 3 | 681 | 1272 | 643 | 831 | 616 |
| Gastric Cancer | 2 | 2201 | 2227 | 356 | 1841 | 199 |
| Colorectal cancer | 5 | 1247 | 4052 | 386 | 2298 | 216 |
| Glioblastoma | 1 | 965 | 1944 | 200 | 763 | 100 |
| head and neck squamous cell carcinoma | 2 | 2459 | 2738 | 467 | 1984 | 333 |
|  |  |  |  |  |  |  |
| Total | 13 |  | 12233 | 840 | 3409 | 534 |

**Table S3- The cell lines in publications used as a data recourse for HCSD**

**SUPPLEMENTARY FIGURES**

| **PUBMED ID** | **#** | **Cell lines** |
| --- | --- | --- |
| 20124221 | 23 cell lines | NPC-TW02, NPC-TW04, NPC-BM1, Colo205, SW480, SW620, SK-Hep-1, HepG2,  Hep-3B, OEC-M1, SCC-4, U1, U4, MCF-7, MDA-MB-435S, C-33A, HeLa, CL1–0, CL1–5, PANC-1, MIA-PaCa-2, A-431 and Jurkat |
| 24681409 | 12 cell lines | HCT116, LoVo, Colo320HSR, WiDr, SW1116, SW620, RKO, DLD1, LS174T, LS180, Colo205 and SW480 |
| 23805888 | 8 cell line | OVTOKO, OVISE, OVMANA, OVSAYO, OVSAHO, OVKATE, RMUG-S and MCAS |
| 23684732 | 2 cell line | LIM1215 and LIM1863 |
| 23443137 | 2 cell lines | KM12SM and KM12C |
| 23483059 | 1 cell line | HNGC-2 |
| 21574646 | 4 cell lines | U87, LN18, T98 and U118 |
| 23591195 | 3 cell lines | EJ28, 5637 and HB-CLS-2 |
| 22918226 | 3 cell lines | FaDu, UTSCC8 and UTSCC42a |
| 22623176 | Tumor Tissue |  |
| 22408128 | 4 cell lines | SW480, SW620, HCT116 and HT29 |
| 21806062 | Malignant pleural effusion |  |
| 19776420 | 4 cell lines | H23, H520, H460 and H1688 |
| 21601021 | 3 cell lines | HNGC2, LN229 and U87MG |
| 21598386 | 4 cell lines | SCC4, SCC38, HSC2 and AMOSIII |
| 21262398 | 1 cell line | A549 |
| 21186846 | 2 cell lines | CL1-0 and CL1-5 |
| 21714544 | 2 cell lines | OEC-M1 and SCC4 |
| 19655816 | 2 cell lines | CL1-0 and CL1-5 |
| 21625447 | 2 cell lines | BxPC-3 and MIA PaCa-2 |
| 22908226 | 5 cell lines | PC3, DU145, LNCaP, 22Rv1, and VCaP |

Label free

Label-based

**Figure S1-**The barplot of the proteins measured in label-free and label-based studies for 16 cancers type and predicted to be secreted by classic or non-classic secretion pathways.

**Label-free**

**Label-based**

**Figure S2-**The barplot of the transmembrane proteins measured in 16 cancers type in total 35 label-free and label-based.
